# Supplementary material for: JAFFA: High sensitivity transcriptome-focused fusion gene detection
Source: Genome Med. 2015 May 11;7(1):43. doi: 10.1186/s13073-015-0167-x (PMC4445815; doi:10.1186/s13073-015-0167-x)
Supplement: Additional file 1: — Includes 12 supporting figures and four supporting tables. A description of each is given within the file. [file 13073_2015_167_MOESM1_ESM.docx]

**Supplementary figures and tables for “JAFFA: High sensitivity transcriptome-focused fusion gene detection”**

**Supplementary figure 1: The JAFFA fusion detection pipeline.** JAFFA encompasses three modes (or pipelines): Assembly, Hybrid and Direct. In Assembly mode the reads are assembled into longer sequences – contigs. In the Direct pipeline the assembled contigs are replaced by the read sequences themselves. To improve computational speed, we first remove duplicate reads and reads mapping to the reference transcriptome. In the hybrid pipeline, we follow the assembly pipeline, then the direct pipeline. The candidates from these two branches are merged prior to final filtering. Figure 1 in the manuscript illustrates some of the steps in the pipeline in more detail.

**
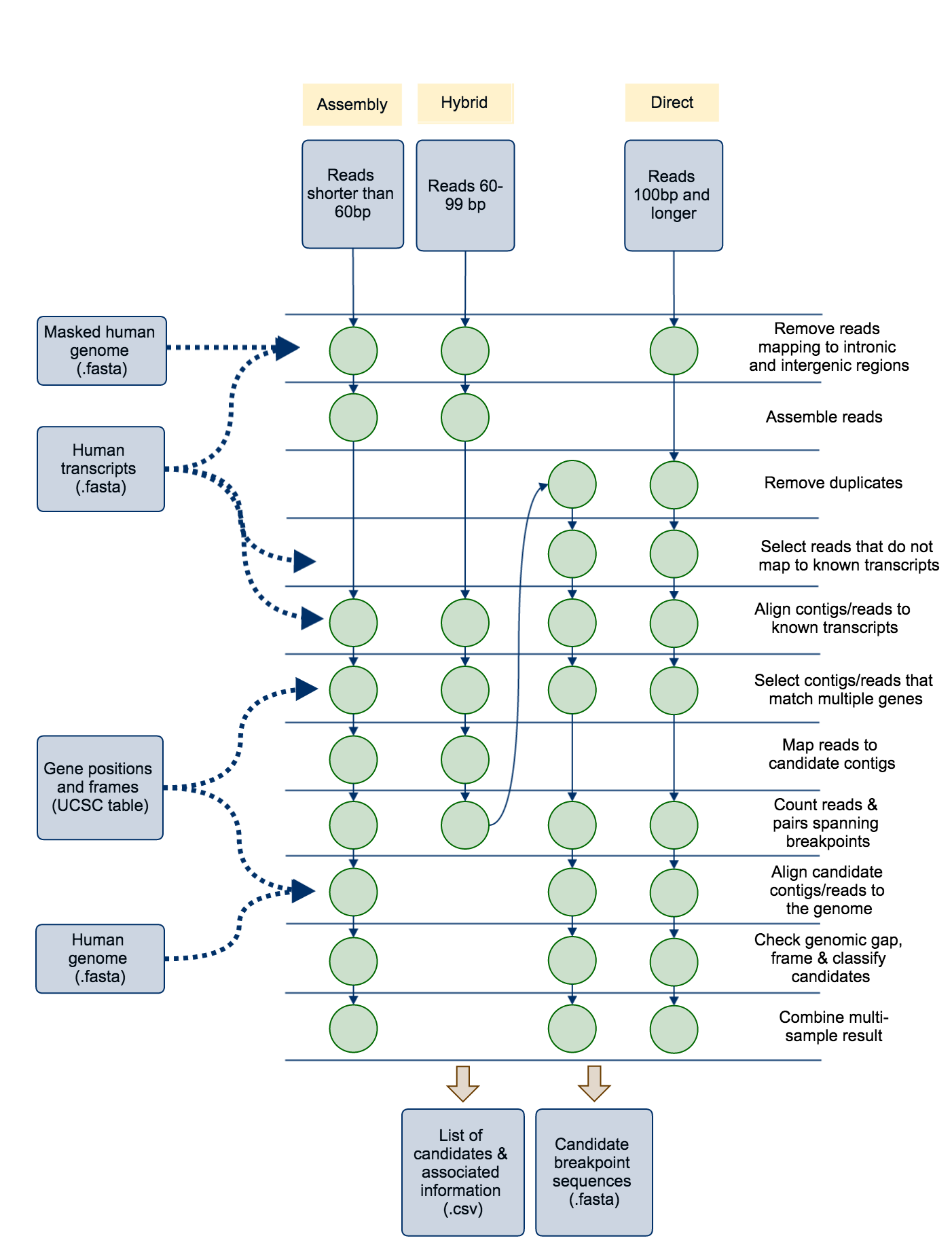
**

**Supplementary material 1: *de novo* assembly**

Choice of *de novo* assembler

Before assessing the JAFFA pipeline as a whole, we first investigated the choice of *de novo* assembler. Both the JAFFA Assembly and Hybrid modes rely on *de novo* assembly and the sensitivity to detect gene fusions is ultimately limited by whether fusion breakpoints are assembled. We tested assemblies produced by Trinity r2013_08_14, Velvet 1.2.10 / Oases 0.2.08, ABySS 1.3.7 / Trans-ABySS 1.4.8 and SOAPdenovo-Trans 1.03 (127mer) on the Edgren dataset (Additional File 2). All tools apart from Trinity were run with *k*-mer lengths of 19, 23, 27, 31 and 35. Settings were kept as default, except that we excluded assembled contigs less than 100bp in length. We used BLAT to identify contigs containing known transcriptional breakpoints with at least 30bp of flanking sequence either side of the breakpoint.

We found that Oases assembled the highest number of known breakpoints (78%), followed by Trans-ABySS (58%), Soapdenovo-Trans (50%) and Trinity (45%). Based on these results, JAFFA incorporates Oases as its default assembler. It should be noted that the assembly properties optimal for fusion detection appear to differ from those for other purposes such as annotation or differential expression. Oases produces more fragmented transcript assemblies, which are often less desirable for other purposes.

Dealing with false chimeras

*De novo* transcriptome assemblies are notorious for producing many false chimeras. False chimeras arise for example, in non-strand specific RNA sequencing, because genes that overlap in the genome cannot be resolved individually. These chimeras will not be detected by JAFFA because there is no breakpoint within a gene. False chimeras may also be constructed during assembly due to homology between gene sequences, such as paralogs, and may be exacerbated by sequencing errors. Assembly involves building a De Bruijn graph from all read subsequences of length *k*. Consequently, genes that share a run of *k* or more bases will share the same node in the De Brujin graph. Traversal of such a graph may produce a false chimera. This effect is easily observed if we look at the frequency of the number of bases shared between genes at the breakpoint of false chimeras (Supplementary figure 2, below). By requiring that the number of bases shared is less than the smallest *k*-mer length (by default we require 13 or fewer) we remove the majority of these events. Any remaining false positives are removed in the same manner as false chimeras arising from other sources, through a series of filtering steps described in Materials and Methods in the manuscript.

**Supplementary figure 2. A large proportion of false chimeras arising from *de novo* assembly have *k* bases or more in common at the break point.**

(A) When two genes share a string of identical bases, assembly may result in a false chimera between the two genes. This typically happens when the length of the shared sequence is the *k*-mer length or longer. The *k*-mer length is a parameter of De Bruijn graph assembly that controls the length that reads are sub-sequenced to. (B) We show the length of shared sequence at the breakpoint for assembled transcripts that match multiple genes – i.e. fusions identified in the first stage of JAFFA prior to other filtering. These preliminary fusion candidates were identified in the BEERS dataset, where no fusions were simulated. The peak at 19 is consistent with the minimum *k*-mer length of 19 used for the assembly. Only candidates with 13 or fewer shared bases (left of dashed line) are forwarded to the next filtering stage of JAFFA (38%). (C) For comparison, we also show the length of shared sequence when reads (Direct mode) are used to identify fusions rather than assembled contigs. No peak is seen around a length of 19 bases.

**Supplementary figure 3: True positive fusions are predominantly local within the genome.** (A) We examined 1884 fusions reported in the Mitelman database (<http://cgap.nci.nih.gov/Chromosomes/Mitelman>), and found that partner fusion genes are commonly located on the same chromosome (44%) and co-localised (19% within 3Mb). Based on these data JAFFA ranks fusions in ascending order of genomic gap size when the spanning reads are equal. See the manuscript for more detail on fusion ranking. Co-localisation of fusions also informs unknown positives (i.e. reported positives other than true or probable true positives) in our validation dataset. (B) False positives in the BEERS simulation are typically not co-localised, whereas in the (C) Edgren, (D) ENCODE and (E) the glioma dataset, many unknown positives are co-localised, suggesting that at least some may be real fusions. Note, these events are unlikely to be run-through transcription because around 70% of events with a genomic distance of < 3Mb involve a non-linear order of genes, suggesting rearrangement.

**Supplementary figure 4: Concordance between MCF-7 datasets.** To the best of our knowledge all MCF-7 sequencing (Edgren, ENCODE and PacBio) was performed on ATCC cell lines. Differences between these three datasets likely occur because of variation in library preparation, sequencing methodology, sequencing depth and because of biological variation in cell lines from different laboratories. The Venn diagrams in (A) and (B) below, show the consistency in fusions predicted by JAFFA, across the MCF-7 Edgren, ENCODE and PacBio datasets. Figure (A) gives the number of true positives, whereas (B) shows all other positives. Most fusion genes are predicted in only one dataset. In (C) we show the number of reads for the Edgren dataset against the ENCODE dataset, for all genes involved in a true positive fusion. These values are shown on a log 2 scale (Pearson correlation=0.89). The correlation in expression for all genes, including those not involved in a fusion, is slightly higher (Pearson correlation=0.92).

**Supplementary table 1: Comparison of fusion detection algorithms.** Here we show results from fusion detection tools we ran as well as results from previous studies that have compared the performance of fusion detection tools using A) simulation from FusionMap and B) RNA-Seq of BT-474, SK-BR-3, KPL-4 and MCF-7. The previous studies counted the rate of detection of the initial 27 fusions identified and validated by Edgren et al. (*Genome Biol* 2011). It should be noted that predictions classed as “Unknown Positives” may have been validated in later studies. Regardless, we found this a useful measure of the sensitivity of various tools. JAFFA appears to have a good balance between sensitivity and the number of candidates reported, as does FusionCatcher, SOAPfuse and deFuse. These are the methods we chose to compare in the manuscript (methods are highlighted in green). Sources: ^1^(Carrara et al., *Biomed Res Int* 2013), ^2^(Carrara et al., *BMC Bioinformatics* 2013), ^3^(Kim & Salzberg, *Genome Biol* 2011) and ^4^(Liu, Ma, Chang, & Zhou, *BMC Bioinformatics* 2013)

**A) FusionMap dataset**

|  | True Positives | Sensitivity | False Positives |
| --- | --- | --- | --- |
| JAFFA - Hybrid | 44 | 88% | 0 |
| FusionFinder | 41^1,2^ | 82%^1,2^ | 10^1,2^ |
| FusionMap | 40^1,2^ | 80%^1,2^ | 3^1^ 6^2^ |
| FusionHunter | 40^1^  20^2^ | 80%^1^ 40%^2^ | 2^1^ 4^2^ |
| MapSplice | 40^1^ 39^2^ | 80%^1^ 78%^2^ | 12^1^ 23^2^ |
| TopHat-fusion | 40^1,2^ 27 | 80%^1,2^ 54% | 39^1^ 73^2^ 0 |
| JAFFA - Assembly | 39 | 78% | 0 |
| SOAPfuse | 37 | 74% | 1 |
| JAFFA - Direct | 34 | 68% | 0 |
| deFuse | 32^1,2^ 34 | 64%^1,2^ 68% | 4^1,2^ 0 |
| Barnacle | 27 | 54% | 0 |
| ChimeraScan | 9^1^ | 18%^1^ | 0^1^ |
| FusionCatcher | Unable to run on a low number of reads | | |

**B) Edgren dataset**

|  | 27 Validated Candidates | Unknown Positives |
| --- | --- | --- |
| TophatFusion | 19^1^ 16^4^ 25^3^ 24 | 136621^1^ 95^4^ 51^3^ 237 |
| SOAPfuse | 24 | 37 |
| FusionQ | 22^4^ | 276^4^ |
| JAFFA - Assembly | 20 | 22 |
| DeFuse | 16^1^ 20^4^ 20 | 899^1^ 1912^4^ 56 |
| ChimeraScan | 19^1^ | 13327^1^ |
| FusionCatcher | 17 | 14 |
| FusionFinder | 13^1^ | 2188^1^ |
| FusionMap | 4^1^ | 65^1^ |
| Barnacle | 9 | 17 |
| FusionHunter | 8^1^ | 18^1^ |

**Supplementary methods 2: fusion tool running parameters**

TopHat-Fusion 2.0.13 was run with the parameters similar to those specified on its example website for analyzing the Edgren dataset. Specifically, using the tophat options “--fusion-search --keep-fasta-order --bowtie1 --no-coverage-search --max-intron-length 100000 --fusion-min-dist 100000 --fusion-anchor-length 13 --fusion-ignore-chromosomes chrM,chrUn_gl000220” and the tophat-fusion-post options “--num-fusion-reads 1 --num-fusion-pairs 2 --num-fusion-both 5”. For single-end reads we set “--num-fusion-pairs 0”. The insert size (-r) and standard deviation (--mate-std-dev) were modified to match each dataset. For the Edgren dataset we used the insert size and standard deviation advised on the TopHat-Fusion website. For the FusionMap, BEERS, MiSeq, ENCODE and gliomas datasets we used an insert size of 8, 100, 0, 50 and 0 respectively and a standard deviation of 20, 100, 100, 50 and 100 respectively. When the ENCODE reads were trimmed to 50bp, we increased the insert size to 150.

JAFFA 1.06, DeFuse 0.6.2, SOAPfuse 1.26 and FusionCatcher 0.99.3d were all run with default settings. For deFuse, we used the results file that had been thresholded on probability. For all tools, samples within a dataset (e.g. Edgren) were run individually and not pooled. More detail can be found in the shell script, Additional file 5.

**Supplementary table 2. A comparison of fusion detection performance on cancer RNA-Seq.**  (A) The Edgren dataset, consisting of between 7 and 21 million 50bp read pairs of the BT-474, SK-BR-3, KPL-4 and MCF-7 cell lines. Using a list of 99 validated fusions in these cell lines, we compared the predictions of JAFFA to TopHat-Fusion, SOAPfuse, deFuse and FusionCatcher. In total, 48 true positives have been reported for this dataset. Predictions not in the list of validated fusions, but involving a promiscuous partner gene (see manuscript for definition), or fusions that were predicted by three or more tools are designated as probable true positives. (B) We compare JAFFA against alternative tools on the ENCODE dataset which consists of 20 million read pairs of MCF-7. Combing the results of all tools, 30 true positives were observed. JAFFA reports more true positives than the other methods. (C) JAFFA’s high sensitivity is also seen on 100bp paired-end dataset from 13 glioma samples for which 31 true positives are known. The samples range from 15 to 35 million read pairs. In parenthesis we show the value at each of JAFFA’s classifications levels: ( high / medium / low) confidence.

A) Edgren breast cancer cell line dataset

|  | True Positives | Probable TPs | Other Positives |
| --- | --- | --- | --- |
| SOAPfuse | 41 | 0 | 19 |
| TophatFusion | 35 | 5 | 221 |
| deFuse | 29 | 2 | 45 |
| JAFFA - Assembly | 28 (24/3/1) | 1 (0/0/1) | 13 (2/3/8) |
| FusionCatcher | 27 | 0 | 4 |
| JAFFA – Assembly  (supported by >1 read) | 26 (24/1/1) | 1 (0/0/1) | 11 (2/1/8) |

B) ENCODE breast cancer cell line dataset

|  | True Positives | Probable TPs | Other Positives |
| --- | --- | --- | --- |
| JAFFA – Direct | 27 (19/8/0) | 6 (3/3/0) | 114 (6/104/4) |
| SOAPfuse | 22 | 2 | 46 |
| JAFFA – Direct  (supported by >1 read) | 21 (19/2/0) | 3 (3/0/0) | 12 (6/2/4) |
| deFuse | 16 | 7 | 97 |
| FusionCatcher | 16 | 2 | 14 |
| TopHat-Fusion | 13 | 3 | 28 |

C) Glioma dataset

|  | True Positives | Probable TPs | Other Positives |
| --- | --- | --- | --- |
| JAFFA – Direct | 30 (30/0/0) | 45 (41/2/2) | 3888 (155/3250/533) |
| JAFFA – Direct  (supported by >1 read) | 30 (30/0/0) | 45 (41/2/2) | 829 (155/141/533) |
| deFuse | 29 | 37 | 632 |
| TopHat-Fusion | 29 | 23 | 256 |
| FusionCatcher | 28 | 41 | 147 |
| SOAPfuse | 22 | 39 | 238 |

**Supplementary figure 5: Concordance between fusion finding tools.** For each of the A) Edgren, B) ENCODE and C) glioma datasets we show the concordance between fusion calls from JAFFA, FusionCatcher, SOAPfuse, DeFuse and TopHat-Fusions. The number of candidates predicted by all tools combined is shown in black/grey and for each tool separately in colour. True positives are differentiated from others by a darker shade. The x-axis shows how many tools reported the fusion. Most candidate fusions are predicted by a single tool (x=1) (note that the y-axis is on a logarithm scale). Of the candidates called by all tools (x=5), almost all are true positives. Candidates that were neither run-through transcription, nor true positive, but predicted by three or more tools (x=3,4,5) were classed as probable true positives. For the Edgren dataset, there were no examples of this, for ENCODE there were two and for the glioma dataset, 46. We speculate that the gliomas had a larger number of unvalidated genuine fusions because the list of true positives only included in-frame fusions.

**Supplementary figure 6: JAFFA’s Computational Performance.** (A) The computational time for a single thread and (B) RAM required to run JAFFA and four other fusion finding tools on the Edgren dataset. JAFFA ran in equal lowest time on all samples, however it consumes more RAM on the two larger samples. Unlike the other tools whose RAM was constant with respect to input bases, JAFFA on 50bp reads performs a *de novo* assembly which scaled with the input bases. On long reads (100bp), we recommend running the Direct mode of JAFFA which has excellent sensitivity and requires comparable resources to other tools. (C,D) Resources required for the ENCODE dataset (20 million 100bp pairs) running a single thread. (E,F) Resources required for the glioma dataset (25 million 100bp pairs on average), when the 13 samples were run in parallel on 13 cores. All jobs were run on a computing cluster with Intel Xeon E3-1240 v3 CPUs.

**Supplementary Figure 7: ROC curve for the different modes of JAFFA on the ENCODE dataset.** JAFFA’s Direct and Hybrid modes perform similarly. Given the high computational cost of the assembly step in the Hybrid mode, we recommend that the Direct mode is always used for reads of 100bp and longer.

**Supplementary Table 3: The number of true positives, probable true positives and other positives reported for the different modes of JAFFA on the ENCODE dataset**. JAFFA’s Hybrid and Direct mode report similar numbers. See Table 2 in the manuscript for more detail. In parenthesis we show the value at each of JAFFA’s classifications levels: ( high / medium / low) confidence.

|  | True Positives | Probable True Positives | Other Positives |
| --- | --- | --- | --- |
| JAFFA – Direct | 27 (19/8/0) | 6 (3/3/0) | 114 (6/104/4) |
| JAFFA – Hybrid | 27 (14/13/0) | 7 (3/4/0) | 127 (4/107/16) |
| JAFFA - Assembly | 17 (8/9/0) | 3 (2/1/0) | 24 (1/9/14) |

**Supplementary Table 4. A comparison of fusion detection performance on glioma samples.**  The glioma dataset was downsampled to depths of 1, 2, 5 and 10 million read pairs per sample. We show the number of true, probable and other positives identified by five fusion detection tools at each depth. In parenthesis we show the value at each of JAFFA’s classifications levels: ( high / medium / low) confidence.

A) 1 million

|  | True Positives | Probable TPs | Other Positives |
| --- | --- | --- | --- |
| JAFFA - Direct | 12 (7/5/0) | 14 (4/10/0) | 161 (12/143/6) |
| deFuse | 6 | 1 | 33 |
| SOAPfuse | 4 | 3 | 4 |
| TophatFusion | 4 | 2 | 10 |
| FusionCatcher | 4 | 1 | 0 |

B) 2 million

|  | True Positives | Probable TPs | Other Positives |
| --- | --- | --- | --- |
| JAFFA – Direct | 19 (12/7/0) | 22 (11/11/0) | 344 (22/308/14) |
| FusionCatcher | 8 | 8 | 1 |
| SOAPfuse | 6 | 6 | 6 |
| deFuse | 6 | 6 | 40 |
| TopHat-Fusion | 5 | 3 | 17 |

C) 5 million

|  | True Positives | Probable TPs | Other Positives |
| --- | --- | --- | --- |
| JAFFA – Direct | 24 (21/3/0) | 29 (19/10/0) | 838 (34/758/46) |
| FusionCatcher | 13 | 16 | 9 |
| SOAPfuse | 11 | 13 | 34 |
| deFuse | 10 | 11 | 112 |
| TopHat-Fusion | 10 | 8 | 46 |

D) 10 million

|  | True Positives | Probable TPs | Other Positives |
| --- | --- | --- | --- |
| JAFFA – Direct | 26 (25/1/0) | 41 (31/8/2) | 1648 (66/1451/131) |
| FusionCatcher | 19 | 26 | 42 |
| SOAPfuse | 19 | 23 | 89 |
| TopHat-Fusion | 16 | 16 | 93 |
| deFuse | 14 | 21 | 215 |

**Supplementary Figure 8: ROC curves for the glioma dataset.** The glioma dataset was downsampled to depths of 1, 2, 5 and 10 million read pairs per sample. The full dataset ranged between 15 and 35 million read pairs per sample. JAFFA reports more true positives at all depths, and performs well in ranking the true positives. Note that the X-axis has been truncated in most instances, so not all fusions are shown.

**Supplementary Figure 9: JAFFA requires less input reads than other fusion finding tools.** ROC curves for JAFFA on 2 million read pairs per sample from the glioma dataset compared to other tools running on 10 million read pairs per sample. FusionCatcher and SOAPfuse perform best, but JAFFA’s performance is not dissimilar, despite having only 1/5th of the input reads.

**Supplementary figure 10. Performance of JAFFA after removing fusions with single read support.** This figure is similar to Figure 2 in the manuscript, however we only show fusions reported by JAFFA which have multi-read support – where the sum of spanning reads and spanning pairs is greater than 1. (A) An ROC-style curve for the ranking of candidate fusions in the Edgren dataset**.** The number of true positives are plotted against the number of other reported positives from a ranked list of fusion candidates. Probable true positives (see manuscript text for detail) are removed. Higher curves indicate a better ranking of the true positives. For each fusion detection tool, we ranked the candidates using the tools own scoring system, or if absent, the supporting data that maximized the area under the curve. (B) On long read data - the ENCODE dataset consisting of 20 million 100bp read pairs of the MCF-7 cell line - JAFFA ranks true positives higher than any other tool. (C) JAFFA’s sensitivity is confirmed on a second long read dataset – 13 glioma samples with read depths varying from 15-35 million 100bp read-pairs. JAFFA identifies 30 of the 31 true positives (total true positives are indicated by the dashed line). Downsampling the data to mimic smaller read depths indicates that JAFFA has excellent sensitivity compared to other tools.

**Supplementary figure 11 and 12: Performance of JAFFA, FusionCatcher, SOAPfuse, deFuse and TopHat-Fusion for different read lengths and layouts – across sequencing depths.** We compared the performance of JAFFA against four other fusion finding tools on the ENCODE data, trimmed to emulate four different read configurations: single-end 50bp, paired-end 50bp, single-end 100bp and paired-end 100bp. Figure 3 of the manuscript shows the number of positives for each configuration for 4 billion bases sequences in total. Here, we show similar figures when the data is subsampled to different depths: 1 billion bases sequenced and 250 million bases sequenced.

**Supplementary figure 11: 1 billion base pairs sequenced**

**Supplementary figure 12: 250 million base pairs sequenced**
